# Botanical Origin of Pesticide Residues in Pollen Loads Collected by Honeybees During and After Apple Bloom

**DOI:** 10.3389/fphys.2019.01069

**Published:** 2019-09-18

**Authors:** Riccardo Favaro, Lisbeth Marie Bauer, Michele Rossi, Luca D’Ambrosio, Edith Bucher, Sergio Angeli

**Affiliations:** ^1^Faculty of Science and Technology, Free University of Bozen-Bolzano, Bolzano, Italy; ^2^Laboratorio Biologico, Agenzia Provinciale per l’Ambiente e la Tutela del Clima, Bolzano, Italy; ^3^Laboratorio Analisi Alimenti, Agenzia Provinciale per l’Ambiente e la Tutela del Clima, Bolzano, Italy

**Keywords:** palynology, pesticide drift, pollen color, colony loss, agricultural landscape, PHQ, multi-residue analysis

## Abstract

Honeybees closely rely on insect-pollinated plants for their survival. Each forager bee displays a tendency of loyalty toward specific plant species during the many daily foraging flights. Due to the ease of collection, pollen loads have been extensively used as a proxy for detection of pesticide residues. Pollen is the main protein food source for colonies, and its contamination has also been addressed as a reason for the colony losses phenomenon. As honeybees fly over a variable but wide range territory, they might collect pollen from both agricultural, urban and wild environments, also displaying considerable preferences in botanical sources between colonies of the same apiary. It is thus difficult to address the source of the pesticide contamination, when pollen is analyzed as a whole. In the current study, a practical and reliable approach has been proposed to narrow down the source of contamination. Pollen loads have been collected from colonies placed in eight locations over large apple orchard extensions in Trentino-South Tyrol region (Italy), during and 2 weeks after apple blossom. The pollen loads have been separated by the color due to the predominant plant species. On each color group, palynology and multi-residual chemical analyses have been performed in parallel. The pollen hazard quotient (PHQ) was used to estimate the risk to honeybees of each color group and of the total collected pollen. Apple and dandelion pollen were the main portions of the first collection, while a greater variety emerged after the apple blossom. Dandelion was always present in the samples. The frequency and the amount of pesticide residues differed according to the collection periods, the locations and the pollen color groups. The amount of insecticide residues increased after the apple blossom, while no difference between the period was found on fungicide residues. The PHQ values were higher after the blossom due to the insecticide contribution, with highest values of 160,000 and 150,000. The variations within samples did not allow to identify a unique source of contamination, whereas it seems that the pollen from plants outside the agricultural areas has as much residues as the pollen from apple orchards.

## Introduction

Pollinating insects, such as honeybees (*Apis mellifera* L.), are a crucial part of ecosystems for their contribution to plant reproduction. While the role of honeybees has been reappraised in view of the contribution of other wild pollinators (Garibaldi et al., 2013), they are still prominent in commercial orchards, since production, fruit growth and fruit durability are affected by pollination and seed development (Garratt et al., 2014). Due to the recent decrease of wild pollinators, insect-pollinated fruit production started to rely on managed honeybee hives (Calderone, 2012). In order to enhance the yield, honeybee hives are brought to fruit tree orchards during the flowering period, and beekeepers play a key role tightly related to fruit growers.

The use of pesticides in integrated crop management practice allows the farmers to protect their yields, but the chemical treatments are well-recognized as one of the main causes affecting pollinators decline (Potts et al., 2016). To combine crop protection and pollinator protection is a big challenge of today’s agriculture. While avoiding spraying of harmful chemicals during the flowering period ensure to prevent the pollinators from direct exposures, feeding on nectar and pollen represent the main source of contamination (Sanchez-Bayo and Goka, 2014).

Pesticides not only affect the target organisms, but also spread in the ecosystems, contaminating soil, water and plants. Residues on plants have been extensively investigated in recent years (Botías et al., 2016; Long and Krupke, 2016; Lentola et al., 2017). Persistent and systemic molecules, such as neonicotinoids, are absorbed and translocated in the entire plant matrix (Sur and Stork, 2003), lately being found also in nectar and pollen (Blacquiere et al., 2012; Böhme et al., 2018).

Pollen contamination has been the subject of many studies as honeybees rely on it for the majority of their protein supply. A study of the annual pollen intake by Keller et al. (2005), reports the amount of collected pollen per hive ranging from 5.6 to 222 kg, depending on the colony size, the food availability and the season length, while other data show an estimated average value of 20 kg (McLellan, 1977). In order to support this large supply, honeybees take continuous surveys of the surrounding territory, making them reliable bioindicators of environmental pollution (Porrini et al., 2002). One particular aspect of the bee ecology is posed by the tendency of each colony to choose the food source autonomously, for which hives of the very same apiary may collect pollens from different plant species at the same time. On the other hand, single honeybees display loyalty to food source during their daily foraging flights, tuning their search on a specific botanical species as long as it continues to provide flowers (Porrini et al., 2002). Thus, a single honeybee tends to gather pollen loads composed of mainly one plant species.

Several studies provided pollen residues data (Škerl et al., 2009; Genersch et al., 2010; Lambert et al., 2012; Kasiotis et al., 2014; Böhme et al., 2018). These studies provide indications of the amount of pesticide residues found in the collected pollens, but there also have been attempts to estimate the hazard of these pollens on honeybee health (Stoner and Eitzer, 2013; Böhme et al., 2018).

The distribution of pesticide in the environment seems to be not equal, as runoff water and airborne movements spread pesticides from agricultural areas to wild plants (Botías et al., 2016). For instance, crops report higher residue levels than the surrounding vegetation (Longley and Sotherton, 1997), but wildflowers too resulted to be an important source of pesticide contaminations (Botías et al., 2015). Residues analysis of the mixed pollen offer a whole picture of the present contaminants, but lack providing the information about their source. Böhme et al. (2018) provided a first attempt of source recognition performing chemical residues of the collected pollen and in parallel performing palynological analyses. However, also in this case pesticide residues could not be attributed to a specific plant species, as pollen samples were analyzed as mixed samples collected by the bees in a specific period.

In the current study, we aimed to fill the gap of the contamination source knowledge, thus analyzing the amount of pesticide residues in pollen loads. After a single collection from honeybees, the pollen loads were divided by color and each group was characterized botanically. Given the overwhelming abundance of apple orchards in the study area, we focused our attention on pesticide residues during and after apple blossom. As no other crops were blooming during the collections, this peculiar situation offered the chance to focus mainly on the pollens collected from apple orchards and the surrounding environments.

## Materials and Methods

### Biological Material

Honeybees [*Apis mellifera* ssp. *carnica* (Pollmann)] were kept in standard 10-frames Dadant-Blatt beehives for nomadic beekeeping. In each beehive, the brood was spread at least over four frames. Bee colonies were managed according to good beekeeping practice and had undergone regular sanitary treatments against the parasitic mite *Varroa destructor* (Anderson & Trueman).

### Field Experimental Setup

The experiments took place in the Italian region Trentino-South Tyrol between April and May 2017. In the lower valleys, this alpine region is characterized by a wide extension of apple orchards composed by many small lots belonging to different farmers (Tasser et al., 2009). Dandelion (*Taraxacum* spp.) is the predominant herbaceous flowering plant on the apple orchard floor during blossom. Its abundance leads to competition with apple trees for pollinators, as already noticed by Free (1968). The woods on the surrounding mountain slopes are characterized mainly by broadleaves species (*Castanea sativa* Mill., *Fraxinus ornus* L., *Quercus pubescens* Willd., *Ostrya carpinifolia* Scop.), and pines (*Pinus sylvestris* L.).

**FIGURE 1:**
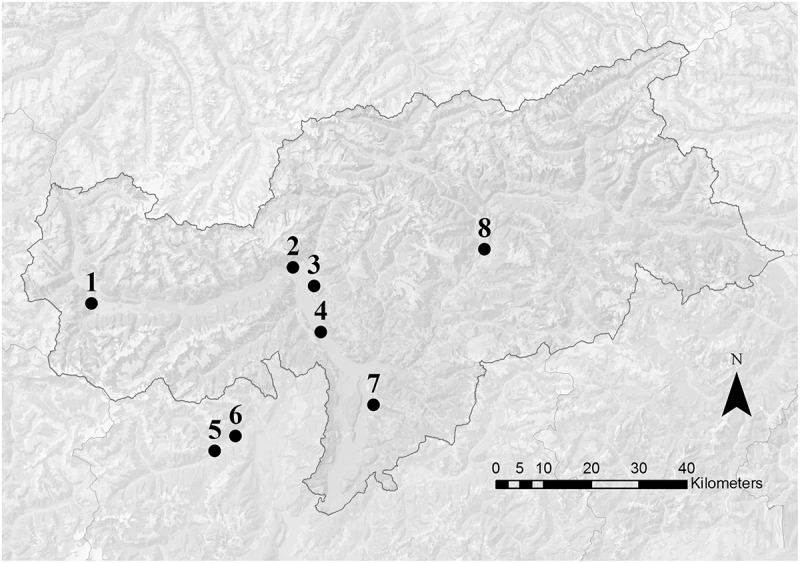
Map of South Tyrol (Italy). Dots indicate the approximate position of the apiary locations. Numbers refer to the information reported in Table 1. Apiaries 5 and 6 were located in the nearby Trento province.

In this region apple trees flower usually in April with temporal shifts of some weeks depending on local conditions. The pollen samples were collected in two periods: during the middle of the main apple blooming (during king flower = F collection) and 2 weeks after the end of the blooming (after flowering = AF collection). Eight voluntary beekeepers participated to the study with their beehives: six apiaries were located along the two main valleys of the province of Bolzano and two apiaries in a valley of the province of Trento (Figure 1). In order to ensure the beekeepers privacy, only altitude and the nearest town are reported for each location (Table 1). Every beekeeper had a permanent apiary within the apple orchard extension in the low valley, in which two beehives were randomly chosen for pollen collection. In each location, the surrounding agricultural landscape was dominated by apple orchards. Even if the use of different cultivar influences slightly timing and number of pesticide treatments, only one common regulation for the apple orchards management exists (AGRIOS, 2017). For this reason, the allowed pesticides were known but not the timing of their application.

**TABLE 1:** Sample collection specifications.

**Location**	**Town**	**Altitude**	**F collection date**	**AF collection date**
1	Sluderno (BZ)	1008	21.-24.4.2017	5.- 8.5.2017
2	Tirolo (BZ)	646	10.-13.4.2017	2.-5.5.2017
3	Merano (BZ)	620	19.-22.4.2017	4.-7-5.2017
4	Nalles (BZ)	345	11.-14.4.2017	3.-6.5.2017
5	Croviana (TN)	902	22.-25.4.2017	11.-14.5.2017
6	Malè (TN)	953	22.-25.4.2017	10.-13.5.2017
7	Laives (BZ)	335	6.-8.4.2017	9.-12.5.2017
8	Bressanone (BZ)	670	22.4-25.4.2017	26.-29.5.2017

*The nearest town to the apiary is reported. The altitude (m a.s.l) refers to the apiary position. Differences in the time between the two collections depend by different duration of apple blossom, due to cultivar and climatic conditions.*

Samples were collected by standard pollen traps (Pollen trap “Metalori,” Il Pungiglione S.C.S., Italy). The two collection periods lasted four consecutive days (Table 1), during which the beekeepers daily gathered, labeled and froze the pollen loads at −20°C daily in sterilized glass jars. Afterward, pollen samples were transported in freezers to the Free University of Bolzano and stored there at −80°C until analysis.

### Palynological Analyses

Pollen samples from every apiary location, collected over a 4 days period, were mixed together to avoid differences linked to the colony. For a first botanical separation, pollen loads were separated by color. The *green* and the *orange* pollen loads were taken out of a representative amount of 50 grams from the *total* fresh pollen loads collected by each beekeeper. The pollen loads were manually separated according to the color (Kirk, 2006) under a neutral light bulb (True-Light led, CRI 96) until reaching samples of at least 10 g each. Once separated, the pollen sample colors were measured in L^∗^a^∗^b color scale with a Chroma meter (Minolta CR-400, Konica Minolta, United States). The abundance of each color in the pollen samples was expressed as a weight percentage over the *total*. The pollen composition of the *total*, *green*, *orange*, and *leftover* samples collected during the flowering, as well as of the *total, orange*, and *leftover* samples collected after the flowering were analyzed by light-microscopy.

The pollen loads of the AF collection were divided by color and every subsample was weighted. From each subsample two pollen loads were randomly chosen for the palynological analysis. The pollen loads were dissolved in 2 ml Milli-Q water and two drops of 60 μl were placed on a microscope slide. After drying, one drop of glycerine jelly with fuchsine (Lanzoni S.r.l, Bologna, Italy) was placed above and covered with a thin glass sheet. Under a microscope, 1000 grains of pollen were counted for each slide and identified based on their morphology at the family, genus or species level, when possible (Bucher, 2004). The taxa found in the slides were assigned to the weight of the corresponding subsamples allowing an estimation of the frequency classes.

The method for the palynological analysis was further implemented in a more accurate and time-saving approach. Performing parallel trials on the same samples with different methods, no difference was found in abundance classes and more biodiversity in rare occurring pollen types. Therefore, for each sample of the F collection, 1 g of pollen was taken and diluted with 30 ml of Milli-Q water and then agitated at 100 rps for 30 min. 1 ml of the solution was further diluted in 6 ml of Milli-Q water and again agitated at 100 rps for 30 min. Two drops of 60 μl were placed on a microscope slide. After drying, one drop of glycerine jelly with fuchsine (Lanzoni S.r.l) was placed above and covered with a thin glass sheet. Under a microscope, 1000 grains of pollen were counted for each slide and identified based on their morphology at the family, genus or species level, when possible (Bucher, 2004). The pollen composition thus referred to the proportion of the number of grains belonging to a level over the total.

We considered the following seven groups for color separation: *total*, *green*, *orange*, and *leftover* pollen loads collected during the apple flowering. *Total*, *orange*, and *leftover* were instead considered in the pollen samples from the second collection period. The *leftover* pollen group referred to a mixture of pollen colors obtained by removing the *green* and the *orange* portions. The pollen groups were kept separated by locations, so that for each location there were seven pollen groups.

### Multi-Residual Pesticides Analyses

A total of 56 pollen samples were chemically analyzed considering the 7 pollen groups per location and the 8 locations above described. Samples of 5 grams each were prepared following the multi-residual QuEChERS (Anastassiades et al., 2003) method (Standard Operating Procedure EN15662:2008). The preparations were then analyzed by GC–MS and LC–MS were used to identify 270 active principles and some of their metabolites. The residues were quantified by standard calibration curves using the Agilent MassHunter Quantitative Analysis Software. The limit of quantification (LOQ) was 0.01 mg/kg for all the substances. When below this limit, the results were not considered in the study.

### Toxicological Evaluation

In order to assess the effects of pesticide residues in pollen on honeybees, we decided to use the Pollen Hazard Quotient (PHQ) proposed by Stoner and Eitzer (2013) dividing the concentration (ppb) of each residue found in the analyses by the known LD_50_ value (μg/bee). As a nurse honeybee can consume up to 9.5 mg of pollen a day (Rortais et al., 2005). Böhme et al. (2018) considered relevant a PHQ of 50. Taking into account a PHQ value of 1,000 would imply the consumption of 1% of the LD_50_ in a day, or 10% after 10 days of average nursing period (Stoner and Eitzer, 2013). A further improvement of the PHQ concept considered the summing of all the PHQs in each pollen sample to reach a total PHQ value (tPHQ) (Böhme et al., 2018).

In this study, we considered tPHQ in order to compare the harm of the different pollen groups and collection periods to the honeybee colonies. The LD50 values were acquired by the US EPA ecotoxicology database (US EPA ECOTOX, 2018), the Agritox database (AGRITOX, 2018) and the Pesticide Properties Database of the University of Hertfordshire (Ppdb., 2018). Both contact and oral doses were considered. In the attempt to rely on a worst-case scenario, the lowest of the available values were chosen to calculate the PHQ values.

### Statistical Analysis

The frequency of the residuals and the tPHQ of the pollen samples were analyzed using the software R (R Core Team, 2018). The effect of the collection period, the color group and the type of pesticide on the number of residues per sample were tested using a Linear Mixed-Effect Model (lme4 package, Bates et al., 2015).

The effect of the pollen type and the collection period on the tPHQ were tested with the same approach. Data were Tukey-transformed to fit a parametric distribution. In both the analysis, the locations were considered as a random effect. All the values reported in the paper are expressed as mean ± standard deviation, when not specified differently. The plots were created using the R package ggplot2 (Wickham, 2016).

## Results

### Botanical Characterization of Pollen During and After Apple Flowering

The chromatic separation of pollen loads allowed to divide four groups: *green*, *orange*, *yellow*, *dark yellow*. Small fractions of other remaining colors were grouped together.

Although some variation between locations, the color composition of the pollen collected during the blossom (F collection) showed that *green* and *orange* were the main groups, being on average 49.1 ± 21 and 30.5 ± 20%, respectively. Together they accounted for the majority of the pollen composition (79.6 ± 10.3%). The other groups presented a more variable incidence: 8.5 ± 9.7% the *yellow*, 4 ± 7.8% the *dark yellow*, 8 ± 3.5% the minor colors. The palynology analyses of the *green* and *orange* components of the F collection confirmed the predominance of apple (*Malus* spp. = 60.9 ± 16.6%) and dandelion (*Taraxacum* spp. = 99.6 ± 0.6%) on the two groups, respectively. The palynology analysis of the *total* pollen composition showed a high occurrence of willow (42.8 ± 25.9%), Asteraceae T-form (dominated by *Taraxacum* spp., 21.3 ± 15.4%) and apple (18.3 ± 16.9%) (Table 2). It was not possible to perform also the palynological analysis of the total pollen samples from the Meran site, as we did not reach a sufficient amount of pollen.

**TABLE 2:** Pollen composition of the total samples collected during apple blossom.

**Location**	**Pollen abundance**			
	**>45%**	**16–45%**	**3–16%**	**<3%**
Sluderno (BZ)	*Salix* sp.	−	Asteraceae T-Form	*Malus/Pyrus* sp. *Viburnum* sp.

Tirolo (BZ)	*Salix* sp.	*Malus/Pyrus* sp.	Asteraceae T-form *Platanus* sp.	*Acer palmatum* Thunb. *Ligustrum/Syringa* sp.

Nalles (BZ)	*Malus/Pyrus* sp.	−	Asteraceae T-form *Fraxinus ornus* L.	*Acer* sp. *Aesculus* sp. Lamiaceae M-form *Ligustrum/Syringa* sp. *Viburnum* sp. Juglandaceae Papaveraceae

Croviana (TN)	−	Asteraceae T-form *Malus/Pyrus* sp.	*Salix* sp. *Viburnum* sp.	*Aesculus* sp. *Ligustrum/Syringa* sp. *Betula* sp.

Malè (TN)	*Salix* sp.	Asteraceae T-form	−	*Malus/Pyrus* sp. *Acer* sp. *Fraxinus ornus* L. *Ligustrum/Syringa* sp. *Quercus* sp.

Laives (BZ)	−	*Salix* sp. *Malus/Pyrus* sp. Asteraceae T-form	−	*Acer* sp. *Ligustrum/Syringa* sp. Brassicaceae *Prunus* sp.

Bressanone (BZ)	*Salix* sp.		Asteraceae T-form *Malus/Pyrus* sp. *Fraxinus ornus* L. Brassicaceae *Aesculus* sp.	Rosaceae *Prunus* sp. Papaveraceae *Viburnum* sp. *Fragaria*/ *Potentilla* form

*Values report the frequency classes of the botanical source over 1000 grains of pollen counted for each slide. Pollens were identified based on their morphology at the family, genus or species level, when possible. Merano is not reported because of lack of sufficient pollen amount for the analyses. While the group Asteraceae T-form in palynological studies comprises more genera from the Asteraceae family, here refers mainly to the genus *Taraxacum*.*

In the second pollen collection (AF), the pollen composition showed an increased variability, with standard deviation easily exceeding the average values. While the *green* group remained the most represented (53.8 ± 35.5%), *dark yellow* reached higher values (19.2 ± 28.4%) similar to *orange* (18.1 ± 21.1%). The *yellow* and the minor colors decreased to 3.3 ± 4.2 and 5.5 ± 5.6%, respectively. The *green* component was no longer represented by apple, but from a variety of other plant species (Table 3), since the palynology analysis revealed traces of *Malus* spp. pollen in only two locations. This was expected, as the apple blossom ended 2 weeks before. The *orange* fraction was still dominated by *Taraxacum* spp. but in a slightly lower amount (81.4 ± 17.2%).

**TABLE 3:** Pollen composition of the total samples collected 2 weeks after the end of apple blossom.

**Location**	**Pollen abundance**				
	**>45%**	**16–45%**	**3–16%**	**<3%**	
Sluderno (BZ)	Asteraceae T form	Poaceae *Lonicera* sp.	*Pinus* sp.	Rhamnaceae	*Fragaria/Potentilla* sp.

				*Salix* sp.	*Allium* sp.
				*Malus/Pyrus* sp.	*Aesculus* sp.
				Rosaceae	*Knautia* sp.
				*Berberis* sp.	Cyperaceae

Tirolo (BZ)	*Quercus* sp.	*Fraxinus ornus* L.	*Trachycarpus* sp.	*Pinus* sp.	*Kolkwitzia* sp.
				*Robinia* sp.	Polygonaceae
				Asteraceae A form	*Weigelia* sp.
				*Viburnum* sp.	*Veronica* sp.
				*Trifolium repens* L.	Apiaceae

Merano (BZ)	*Trachycarpus* sp.	−	Ranuncolaceae	*Ilex* sp.	Rhamnaceae
			*Quercus ilex*	*Viburnum* sp.	*Acer* sp.
			Asteraceae T form	*Malus/Pyrus* sp.	*Pinus* sp.
			*Robinia pseudoacacia* L.	*Liriodendron* sp.	*Weigelia* sp.
				*Aesculus* sp.	Geraniaceae
				*Lonicera* sp.	*Tulipa* sp.

Nalles (BZ)	*Gleditsia* sp.	−	*Robinia pseudoacacia* L.	Ranuncolaceae	*Pinus* sp.
			*Trachycarpus* sp.	Asteraceae T form	*Allium* sp.
			*Acer* sp.	*Aesculus* sp.	*Trollius* sp.
			*Cornus* sp.	*Thalictrum* sp.	*Aruncus* sp.
			*Liriodendron* sp.		
			Brassicaceae		

Croviana (TN)	−	*Fraxinus ornus* L.	Brassicaceae	*Pinus* sp.	Apiaceae
		*Quercus* sp.		*Salix* sp.	*Clemantis* sp.
		*Asteraceae T form*			

Malè (TN)	*Fraxinus ornus* L.	−	Asteraceae T form	*Pinus* sp.	Geraniaceae
			*Malus/Pyrus* sp.	*Picea* sp.	Caryophillaceae
				*Acer* sp.	*Knautia* sp.
				*Prunus* sp.	

Laives (BZ)	Asteraceae T form	−	*Gleditisia* sp.	*Cornus* sp.	*Anemone* sp.
			*Trachycarpus* sp.	*Aesculus* sp.	*Viburnum* sp.
			*Liriodendron* sp.	*Rubus* sp.	*Pinus* sp.
			*Fraxinus ornus*	*Acer* sp.	

Bressanone (BZ)	*Parthenocissus* sp.	−	*Rubus* sp.	*Plantago* sp.	Papaveraceae
			*Tilia* sp.	*Potentilla/Fragaria* sp.	*Echium* sp.
			Rosaceae	Asteraceae H form	Lamiaceae
				Asteraceae T form	*Knautia* sp.
				Poaceae	*Fraxinus ornus*
				*Vitis* sp.	*Aruncus* sp.

*Values report the frequency classes of the botanical source expressed as weight proportion over the total. Pollens were identified based on their morphology at the family, genus or species level, when possible. While the group Asteraceae T-form in palynological studies comprises more genera from the Asteraceae family, here refers mainly to the genus *Taraxacum*.*

The variation of pollen composition in the main color groups revealed also a change in the chromametric scale (L^∗^a^∗^b) although these differences were not detected at operator sight. The *green* group, identified as 57.6, 0.3, 32.7 in the F collection became 51.3, 0.8, 27.8 in the AF collection. The *orange* group shifted from 54.8, 18.6, 39.3 in the F collection to 51.3, 18.8, 35.1 in the AF collection.

### Pesticide Residues in Pollen Samples

**FIGURE 2:**
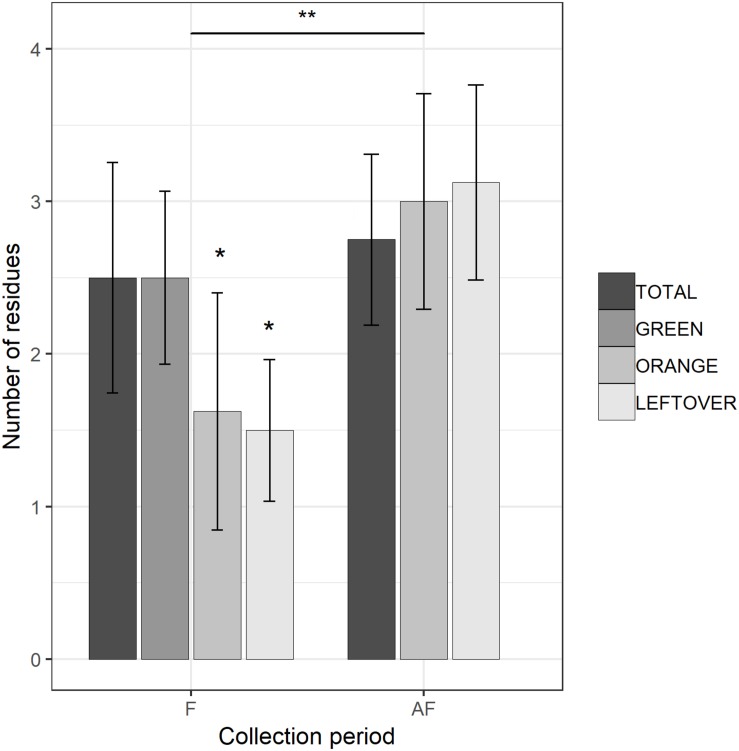
Number of insecticide residues detected in each sample, during (F) and 2 weeks after the end of apple blossom (AF). Asterisks show statistical significance (^∗^*p* < 0.05, ^∗∗^*p* < 0.01). Error bars report standard error of the mean. Pollen color groups are reported in scale of grays.

**FIGURE 3:**
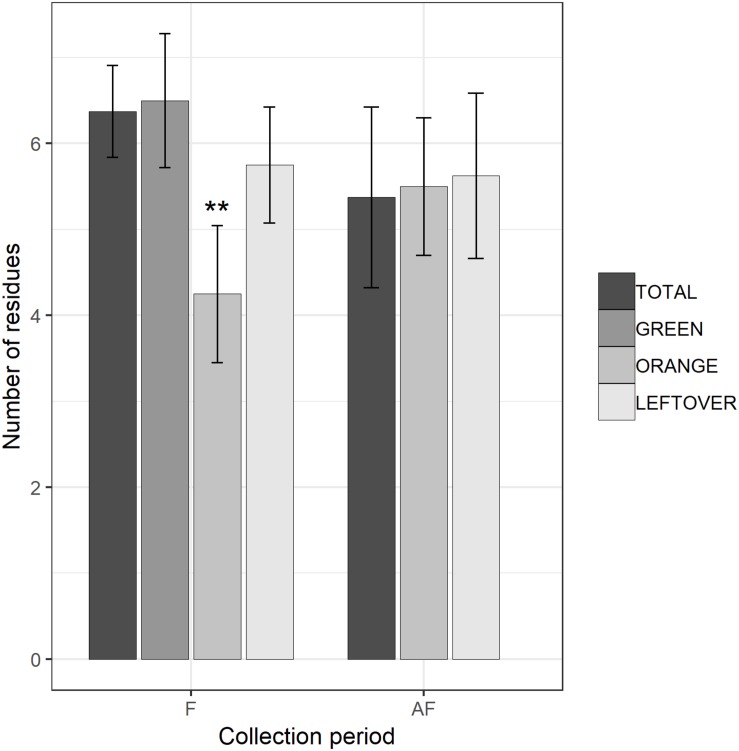
Number of fungicide residues detected in each sample, during (F) and 2 weeks after the end of apple blossom (AF). Asterisks show statistical significance (^∗∗^*p* < 0.01). Error bars report standard error of the mean. Pollen color groups are reported in scale of grays.

The multi-residual analysis on pollen loads from eight locations, collected in two periods, was performed on a total of 56 samples. 36 pesticides were detected considering all samples, of which 13 insecticides, 21 fungicides, and 2 herbicides (Table 4). Overall, fungicides occurred the most, as in each sample we found from 2 up to 10 substances, while insecticides ranged from 0 to 6 residues per sample. No sample was free of pesticides. The statistical analysis of the number of residues showed a higher occurrence of fungicides in the samples (lmer, *t* = −3.05, *p* < 0.005), with an average difference of 2.7 substances per sample from the insecticides (Table 5). The collection period had a highly significant effect on the insecticide residues (lmer, *t* = −2.871, *p* < 0.01) (Figure 2), as their number increased of 1.375 residues per sample during the second collection (AF). On the contrary, there was no effect of the collection period on the number of fungicides per sample (lmer, *t* = −1.39, *p* = 0.17) (Figure 3). The number of fungicide residues in *orange* pollen was significantly lower than in the *total* and in the *green* pollens during the F collection (lmer, *t* = −3.38, *p* < 0.005), as well as the number of insecticides (lmer, *t* = −2.13, *p* < 0.05). After flowering, the number of residues in *orange* pollen raised significantly in insecticides (lmer, *t* = 5.29, *p* < 0.01) but not in fungicides (lmer, *t* = 0.661, *p* = 0.52). Also the *leftover* samples showed a reduced occurrence of insecticide residues during the apple blossom (lmer, *t* = −2.43, *p* < 0.05).

**TABLE 4:** Summary of the 36 pesticides detected in honey bee pollen loads collected during and after apple blossom and separated by color.

**Pesticide**	**Type**	**Period**	**Group**	**Mean ± SD**	**Maximum**	**Frequency**	**LD_50_ contact**	**LD_50_ oral**	**PHQ_max_**
Boscalid	Fg	F	Total	0.062	0.062	1	200 b	160 b	0.3875
			Green	0.1	0.1	1			0.625
			Orange	0.04	0.04	1			0.25
			Leftover	–	–	–			–
					
		AF	Total	0.016	0.016	1			0.1
			Orange	0.02	0.02	1			0.125
			Leftover	0.052	0.052	1			0.325

Bupirimate	Fg	F	Total	0.384 ± 0.41	0.088	2	50 b	200 b	1.76
			Green	0.198 ± 0.25	0.49	3			9.8
			Orange	0.078	0.078	1			1.56
			Leftover	–	–	–			–
					
		AF	Total	–	–	–			–
			Orange	–	–	–			–
			Leftover	–	–	–			–

Captan	Fg	F	Total	1.103 ± 1.7	3.1	3	200 b	100 b	31
			Green	0.17	0.17	1			1.7
			Orange	0.15	0.15	1			1.5
			Leftover	0.26 ± 0.5	1.3	6			13
					
		AF	Total	–	–	–			–
			Orange	0.47 ± 0.62	1.4	4			14
			Leftover	0.22 ± 0.29	0.69	5			6.9

Chlorpyrifos-ethyl	Is	F	Total	–	–	–	0.01 a	0.25 a	–
			Green	–	–	–			–
			Orange	0.02	0.02	1			2000
			Leftover	–	–	–			–
					
		AF	Total	0.76 ± 0.25	0.94	2			94000
			Orange	0.98 ± 0.73	1.5	2			150000
			Leftover	1.1 ± 0.7	1.6	2			160000

Chlorpyrifos-methyl	Is	F	Total	0.13 ± 0.13	0.23	2	0.38	0.11	2090
			Green	0.076	0.076	1			690
			Orange	0.034	0.034	1			309
			Leftover	0.028	0.028	1			254
					
		AF	Total	0.12 ± 0.05	0.15	4			1363
			Orange	0.66 ± 1.1	2.27	4			20636
			Leftover	0.16 ± 0.1	0.24	4			2181

Cyflufenamid	Fg	F	Total	–	–	–	100 c	100 c	–
			Green	0.03	0.03	1			0.3
			Orange	–	–	–			–
			Leftover	–	–	–			–
					
		AF	Total	0.011	0.011	1			0.11
			Orange	–	–	–			–
			Leftover	0.011	0.011	1			0.11

Cyprodinil	Fg	F	Total	0.241 ± 0.4	1.1	7	100 b	150 b	11
			Green	0.31 ± 0.51	1.5	8			15
			Orange	0.1 ± 0.13	0.38	7			3.8
			Leftover	0.15 ± 0.28	0.69	7			6.9
					
		AF	Total	–	–	–			–
			Orange	–	–	–			–
			Leftover	0.043	0.043	1			0.43

Difenoconazole	Fg	F	Total	0.067	0.067	1	100 b	177 b	0.67
			Green	0.16	0.16	1			1.6
			Orange	0.06	0.06	1			0.6
			Leftover	0.062	0.062	1			0.6
					
		AF	Total	0.1 ± 0.12	0.38	5			3.8
			Orange	0.1 ± 0.15	0.34	4			3.4
			Leftover	0.1 ± 0.12	0.28	4			2.8

Dithianon	Fg	F	Total	0.39 ± 0.45	0.71	2	100 b	25.4 b	28
			Green	–	–	–			–
			Orange	–	–	–			–
			Leftover	–	–	–			–
					
		AF	Total	–	–	–			–
			Orange	0.05	0.05	2			2
			Leftover	–	–	–			–

Dodina	Fg	F	Total	0.1	0.12	2	100 c	200 c	1.2
			Green	0.32 ± 0.26	0.51	2			5.1
			Orange	0.1	0.12	2			1.2
			Leftover	0.07 ± 0.1	0.16	3			1.6
					
		AF	Total	0.04	0.08	3			0.8
			Orange	0.026	0.026	1			0.2
			Leftover	0.02	0.02	4			0.2

Etofenprox	Is	F	Total	0.02	0.02	2	0.015 b	0.024 b	1333
			Green	0.11	0.11	2			7333
			Orange	0.016	0.016	1			1066
			Leftover	0.02 ± 0.02	0.046	3			3066
					
		AF	Total	–	–	–			–
			Orange	–	–	–			–
			Leftover	–	–	–			–

Fenoxycarb	Is	F	Total	–	–	–	100 b	204 b	–
			Green	–	–	–			–
			Orange	–	–	–			–
			Leftover	–	–	–			–
					
		AF	Total	–	–	–			–
			Orange	–	–	–			–
			Leftover	0.046	0.046	1			0.46

Flonicamid	Is	F	Total	0.02 ± 0.01	0.06	7	100 b	60.5 b	1
			Green	0.03 ± 0.02	0.1	7			1.6
			Orange	0.02	0.03	4			0.5
			Leftover	0.04 ± 0.03	0.08	5			1.3
					
		AF	Total	–	–	–			–
			Orange	–	–	–			–
			Leftover	–	–	–			–

Fluazinam	Fg	F	Total	0.13 ± 0.1	0.29	7	200 b	100 b	2.9
			Green	0.15 ± 0.11	0.32	6			3.2
			Orange	0.11 ± 0.1	0.23	5			2.3
			Leftover	0.05 ± 0.03	0.08	5			0.8
					
		AF	Total	0.36 ± 0.17	0.68	7			6.8
			Orange	0.47 ± 0.4	1.1	8			11
			Leftover	0.52 ± 0.3	0.87	7			8.7

Fludioxonil	Fg	F	Total	–	–	–	100 b	100 b	–
			Green	0.018	0.018	1			0.18
			Orange	–	–	–			–
			Leftover	0.021	0.021	1			0.21
					
		AF	Total	–	–	–			–
			Orange	–	–	–			–
			Leftover	–	–	–			–

Folpet	Fg	F	Total	0.2	0.2	1	200 b	236 b	1
			Green	0.15	0.15	1			0.7
			Orange	–	–	–			–
			Leftover	–	–	–			–
					
		AF	Total	0.2 ± 0.1	0.32	5			1.6
			Orange	0.43 ± 0.4	1.1	6			5.5
			Leftover	0.3 ± 0.2	0.65	5			3.25

Imidacloprid	Is	F	Total	0.041	0.041	1	0.0439 a	0.0039 a	10512
			Green	–	–	–			–
			Orange	–	–	–			–
			Leftover	–	–	–			–
					
		AF	Total	0.06 ± 0.05	0.18	7			46153
			Orange	0.068 ± 0.03	0.11	6			28205
			Leftover	0.094 ± 0.1	0.32	7			82051

Indoxacarb	Is	F	Total	–	–	–	0.07 b	0.194 b	–
			Green	–	–	–			–
			Orange	–	–	–			–
			Leftover	–	–	–			–
					
		AF	Total	–	–	–			–
			Orange	–	–	–			–
			Leftover	0.085	0.085	1			1214

MCPA	He	F	Total	0.029	0.029	1	200 c	200 c	0.1
			Green	–	–	–			–
			Orange	0.04 ± 0.05	0.1	3			0.5
			Leftover	–	–	–			–
					
		AF	Total	–	–	–			–
			Orange	0.01	0.01	2			0.05
			Leftover	–	–	–			–

Metamitron	He	F	Total	–	–	–	100 c	97.2 c	–
			Green	–	–	–			–
			Orange	–	–	–			–
			Leftover	–	–	–			–
					
		AF	Total	0.06	0.07	2			0.72
			Orange	0.09 ± 0.1	0.22	3			2.2
			Leftover	0.02 ± 0.01	0.03	2			0.3

Methoxyfenozid	Is	F	Total	0.3 ± 0.05	0.34	2	100 a	100 b	3.4
			Green	0.5 ± 0.3	0.79	2			7.9
			Orange	0.2 ± 0.26	0.45	2			4.5
			Leftover	–	–	–			–
					
		AF	Total	0.03 ± 0.01	0.042	4			0.4
			Orange	0.03 ± 0.01	0.04	4			0.4
			Leftover	0.047	0.052	3			0.4

Metrafenon	Fg	F	Total	–	–	–	100 b	114 b	–
			Green	–	–	–			–
			Orange	–	–	–			–
			Leftover	–	–	–			–
					
		AF	Total	–	–	–			–
			Orange	0.021	0.021	1			0.2
			Leftover	–	–	–			–

Myclobutanil	Fg	F	Total	–	–	–	33.9 c	33.9 c	–
			Green	0.03 ± 0.01	0.046	3			1.3
			Orange	–	–	–			–
			Leftover	–	–	–			–
					
		AF	Total	0.09	0.09	1			2.6
			Orange	–	–	–			–
			Leftover	0.034	0.034	1			1

Paclobutrazol	Fg	F	Total	–	–	–	40 b	2 b	–
			Green	–	–	–			–
			Orange	–	–	–			–
			Leftover	–	–	–			–
					
		AF	Total	0.014	0.014	1			7
			Orange	–	–	–			–
			Leftover	–	–	–			–

Penconazole	Fg	F	Total	0.12 ± 0.04	0.35	8	30 b	112 b	11.6
			Green	0.2 ± 0.16	0.57	8			19
			Orange	0.2 ± 0.3	0.8	7			26
			Leftover	0.06 ± 0.04	0.15	8			5
					
		AF	Total	0.05 ± 0.04	0.1	5			3.3
			Orange	0.05 ± 0.02	0.075	5			2.5
			Leftover	0.06 ± 0.04	0.14	5			4.6

Penthiopyrad	Fg	F	Total	0.25 ± 0.47	1.1	5	500 b	500 b	2.2
			Green	0.38 ± 0.6	1.3	4			2.6
			Orange	0.7	0.7	1			1.4
			Leftover	0.26 ± 0.3	0.5	2			1
					
		AF	Total	0.12 ± 0.17	0.5	7			1
			Orange	0.14 ± 0.14	0.4	6			0.8
			Leftover	0.18 ± 0.26	0.74	7			1.5

Phosmet	Is	F	Total	0.175 ± 0.25	0.47	3	1.06 a	0.15 a	3133
			Green	0.17 ± 0.1	0.24	2			1600
			Orange	0.072	0.072	1			500
			Leftover	0.033	0.033	1			220
					
		AF	Total	0.48 ± 0.13	0.62	4			4133
			Orange	0.52 ± 0.4	1.14	5			7600
			Leftover	0.09 ± 0.7	2.1	5			14000

Pirimicarb	Is	F	Total	0.036	0.036	1	12.56 a	4 b	9
			Green	0.05	0.05	1			12.5
			Orange	0.036	0.036	1			9
			Leftover	–	–	–			–
					
		AF	Total	–	–	–			–
			Orange	–	–	–			–
			Leftover	–	–	–			–

Pyraclostrobin	Fg	F	Total	0.053	0.053	1	100 b	73.1 b	0.7
			Green	0.074	0.074	1			1
			Orange	0.015	0.015	1			0.2
			Leftover	0.06 ± 0.03	0.082	2			1.1
					
		AF	Total	0.011	0.011	1			0.2
			Orange	–	–	–			–
			Leftover	–	–	–			–

Pyrimethanil	Fg	F	Total	0.2 ± 0.2	0.5	4	100 b	100 b	5
			Green	0.37 ± 0.42	0.97	4			9.7
			Orange	0.2 ± 0.2	0.52	4			5.2
			Leftover	0.18 ± 0.12	0.3	3			3
					
		AF	Total	0.11 ± 0.11	0.23	3			2.3
			Orange	0.07 ± 0.05	0.13	3			1.3
			Leftover	0.16 ± 0.07	0.22	2			2.2

Quinoxyfen	Fg	F	Total	0.05 ± 0.04	0.12	4	100 b	100 b	1.2
			Green	0.1 ± 0.1	0.25	4			2.5
			Orange	0.041	0.041	1			0.4
			Leftover	0.05 ± 0.05	0.11	3			1.1
					
		AF	Total	0.02	0.02	2			0.2
			Orange	0.13 ± 0.14	0.29	3			2.9
			Leftover	0.02 ± 0.01	0.042	3			0.4

Spirotetramat	Is	F	Total	–	–	–	100 a	107 a	–
			Green	–	–	–			–
			Orange	–	–	–			–
			Leftover	–	–	–			–
					
		AF	Total	–	–	–			–
			Orange	–	–	–			–
			Leftover	0.01	0.01	1			0.1

Tau-fluvalinate	Is	F	Total	0.13 ± 0.1	0.21	2	12 b	12.6 b	17.5
			Green	0.4 ± 0.72	1.5	4			125
			Orange	0.4	0.4	1			33
			Leftover	0.08	0.08	1			6
					
		AF	Total	0.01	0.01	1			0.8
			Orange	0.03 ± 0.02	0.05	2			4
			Leftover	0.015	0.015	1			1

Tetraconazole	Fg	F	Total	0.11 ± 0.1	0.18	2	63 b	130 b	2.8
			Green	0.4 ± 0.6	1.1	3			17.5
			Orange	0.017 ± 0.2	0.02	3			0.3
			Leftover	0.02 ± 0.01	0.03	5			0.5
					
		AF	Total	0.011	0.011	1			0.2
			Orange	–	–	–			–
			Leftover	–	–	–			–

Thiacloprid	Is	F	Total	–	–	–	37.83 a	17.32 b	–
			Green	0.024	0.024	1			1.4
			Orange	0.11	0.11	1			6.3
			Leftover	–	–	–			–
					
		AF	Total	–	–	–			–
			Orange	–	–	–			–
			Leftover	–	–	–			–

Trifloxystrobin	Fg	F	Total	0.1	0.1	1	200 b	200 b	0.5
			Green	–	–	–			–
			Orange	–	–	–			–
			Leftover	–	–	–			–
					
		AF	Total	–	–	–			–
			Orange	–	–	–			–
			Leftover	–	–	–			–

*Type reports the pesticide type according to Pesticide Properties DataBase (Fg = fungicide; Is = insecticide; He = herbicide). Concentration values are expressed in ppm (mg/Kg). PHQ has been calculated as pesticide residue concentration in ppb/LD50. Letters indicate the sources for LD50 values: (a) US EPA ECOTOX database. (b) EU AGRITOX database. (c) Pesticide Properties DataBase.*

**TABLE 5:** Number of pesticides detected in pollen samples from different color group, during and after apple blossom, for each location.

**Location**	**Collection**	**n° insecticides**					**n° fungicides**				
		**Total**	**Green**	**Orange**	**Leftover**	**Mean**	**Total**	**Green**	**Orange**	**Leftover**	**Mean**
1	F	1	3	1	1		5	6	3	6	
2	F	1	3	2	0		5	4	2	2	
3	F	4	5	3	2		8	9	4	6	
4	F	5	6	6	4		8	9	9	8	
5	F	1	2	1	1		5	9	3	7	
6	F	1	2	1	2		8	6	6	7	
7	F	1	2	1	0		5	5	3	6	
8	F	6	5	6	2		7	4	4	4	
		
	Average	2,5	3,5	2,6	1,5	2,53	6,4	6,5	4,3	5,75	5,72
	Sum	20	28	21	12		51	52	34	46	

1	AF	2		2	3		3		2	4	
2	AF	4		6	3		5		6	6	
3	AF	5		6	5		8		8	8	
4	AF	3		5	5		8		7	8	
5	AF	2		3	2		5		4	5	
6	AF	2		3	2		5		3	6	
7	AF	0		1	0		0		6	0	
8	AF	4		6	5		9		8	8	
		
	Average	2,75	−	4	3,125	3,29	5,375		5,5	5,625	5,50
	Sum	22	−	32	25		43		44	45	
		
	Mean					2,91					5,61

*Values indicate the number of insecticide or fungicide residues found in pollen samples. The average of each color group is reported on the “average” row. The sum of the number of residues of each color group is reported on the “sum” row. The mean of the number of all the insecticide and fungicide residues is reported on the “mean” label.*

The presence and the amount of residues varied according to the collection period and the pollen group. Among the insecticides, Flonicamid, Phosmet, Imidacloprid, Methoxyfenozid and Chlorpyrifos-methyl were the most frequent, being found in 23, 21, 21, 17, and 17 samples, respectively. Penconazole, Cyprodinil, and Fluazinam were instead the most frequent fungicides (in 50, 38, and 31 samples, respectively). We detected only two herbicides, Metamitron in six samples, and MCPA in six samples (Table 4).

The analysis revealed the highest concentrations of insecticides in the AF collection, where Chlorpyrifos-methyl and Phosmet reached 2.27 and 2.1 ppm respectively (Table 4). Some substances were present only in the F collection (Etofenprox, Flonicamid), while others appeared after the flowering (Indoxacarb, Fenoxycarb, Spirotetramat) (Table 4). We also found residues of insecticides classified harmful to bees in the F collection: Chlorpyrifos-ethyl and Imidacloprid were found at the maximum concentration of 0.2 ppm (*orange* pollen) and 0.041 ppm (*total* pollen), respectively.

### Pollen Hazard Quotient

In the attempt to evaluate the toxicological effect of the residues found on pollen to adult honeybees, we calculated the PHQ for each substance found in a sample and then we summed them together in tPHQ (Stoner and Eitzer, 2013; Böhme et al., 2018). The PHQ value depends on the residues concentration and the substance LD_50_. We found the highest values of PHQ in insecticide residues, where Chlorpyrifos-ethyl reached a PHQ of 160,000 in an AF *leftover* sample (Table 4). As 1,000 PHQ accounts for 1% of the LD50 consumed by a nurse bee in a day, this value of Chlorpyrifos-ethyl means a consumption of 160% of the LD50 in a day. Other high PHQ resulted from Imidacloprid (28,205), Chlorpyrifos-methyl (20,636), Phosmet (7,600) and Chlorpyrifos-ethyl again (150,000) all in *orange* AF samples (Table 4). The highest Imidacloprid PHQ was 82,051 from a *leftover* pollen sample as well as the highest Phosmet PHQ (14,000). Fungicides usually have higher LD_50_, thus lower PHQs. We found Captan having the higher PHQ of 31 (*total* pollen, F collection), followed by Dithianon (27.95 in *total* pollen, F collection) and Penconazole (26.67 in *orange* pollen, F collection). Overall, the mean PHQ of fungicides (2,55 ± 7.02) was much lower than the PHQ of insecticides (6,080 ± 19,030). The PHQ of the herbicides was negligible, having a mean of 0.4 ± 0.57, with Metamitron reaching a maximum value of 2.26. The insecticide residues accounted for the 74 ± 40% of the tPHQ in the samples. In 69% of the samples, the insecticide contribution was higher than 90%.

**FIGURE 4:**
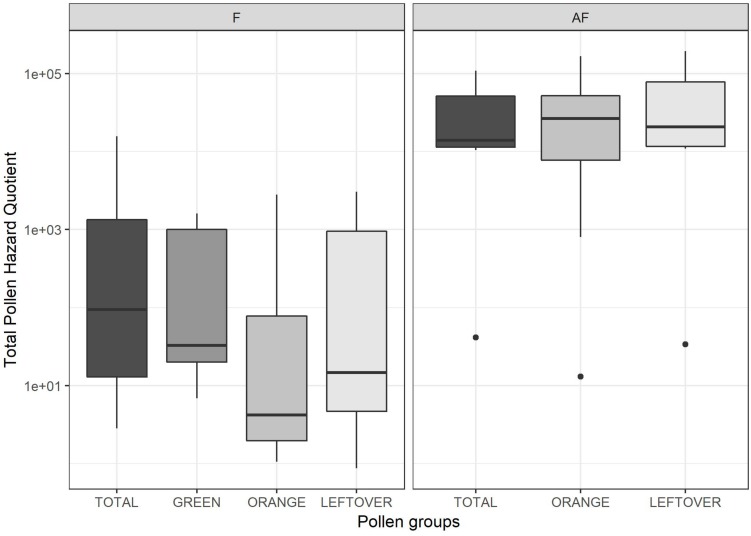
Total Pollen Hazard Quotient (PHQ) of the pesticide residues detected in each sample, during (on the left frame, F) and 2 weeks after apple blossom (on the right frame, AF). Values are reported on a logarithmic scale. Dots indicate outliers.

**FIGURE 5:**
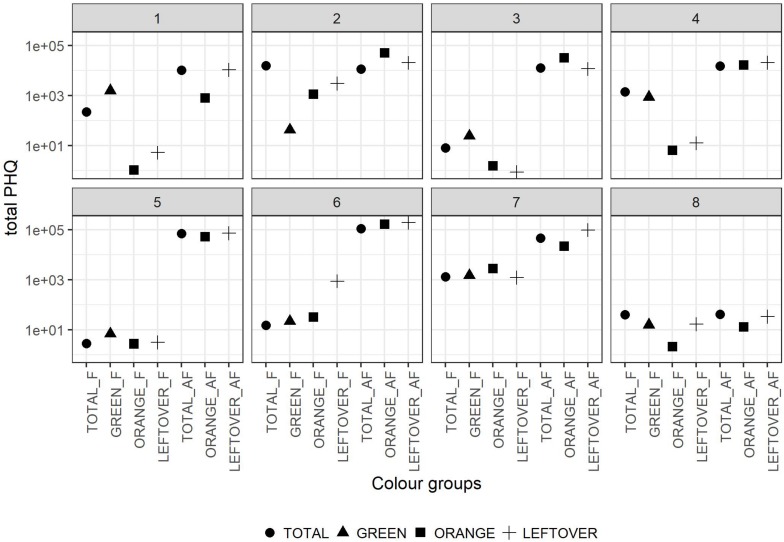
Total PHQ of the pollen color groups in the eight locations. Dots shapes refer to the pollen color group. The values in y-axis are reported in logarithmic scale. In the x-axis the labels combine the pollen group and the collection period (F = apple flowering, AF = after apple flowering).

The tPHQs varied according to the collection period and pollen group (Figure 4). In the pollen collected during the apple blossom, the mean tPHQs resulted in 2,354 ± 5,455 for *total*, 498 ± 1,013 for *orange*, 509 ± 703 for *green* and 651 ± 1,089 for *leftover* pollen samples. The values significantly increased in the AF collection (lmer, *t* = −6.446, *p* < 0.001), where the *total* pollen scored 39,525 ± 38,244, the *orange* pollen 42,787 ± 52,824 and the *leftover* pollen 61,082 ± 67,241 (Figure 4). Due to the high data variability, it was not possible to detect statistical differences between pollen groups, although consistent variations were found. However, pollen color groups collected from the same apiary showed remarkable differences in terms of tPHQ (Figure 5).

Since the purpose of the experiment was to detect the pesticides contribution of different pollen groups, we did not test the pollen toxicological effects, nor did we monitor the colonies health. However, none of the beehives involved in the experiment collapsed by the end of the trial.

## Discussion

This study provides for the first time insight into the contribution of plant specific residues in bee-collected pollen. The study was performed in a context of large apple orchard extensions in Trentino-South Tyrol (Italy). Our results clearly showed the source of pesticide contaminations according to the separation of pollen loads by their color, then assessing the number of residues and their potential toxicity. Here we combined two well-known practices, palynology and multi-residual analysis, with the purpose to obtain a more detailed picture of the agricultural landscape effect on pesticide contamination. The color of the pollen loads successfully allowed to separate the main plant species pollen during and after apple blossom, apple and dandelion, from the pollen of the surrounding plants. Although the pollen analysis revealed that not all the pollen of a giving color group belonged to a single species, this single species represented the main fraction, in particular in the *orange* group, which was almost totally composed by dandelion (99% during F collection, Table 2). *Green* pollen had a lower amount of apple pollen, but still representing 61%. The remaining received contributions by other green pollen species, such as *Salix* spp., which color ranges from yellowish green to full green (Kirk, 2006).

It was surprising that *Salix* spp. (42.8%) was the most abundant pollen during the F collection, even if the hives were placed in the midst of full blooming apple orchards. Indeed, dandelion and apple were only 21.3 and 18.3% of the collected pollen loads. This finding not only confirmed the studies of Free (1996, 1968), addressing dandelion as a competitor of apple flowers, but also showed that other plants from the surroundings (e.g., willows) might exhibit an even stronger appeal. Mitchell et al. (2009) suggested an effect of the nectar and pollen availability on flowers due to temperature, and the constancy of bees on one flower species. Similar results were found when hives were placed in the center of a large apple commercial production providing only 25% of apple pollen while willow reached almost 60% (Olsen et al., 1979).

While the main two components during the F collection were the *green* and the *orange* (79.6%), the lack of apple flowers in the AF collection led to increased variability of the other pollens. The *orange* component dropped from 30.5 to 18%, and other plant species from the surrounding environment became the most abundant. These plants, such as *Fraxinus ornus*, *Gleditisia* sp., *Parthenocissus* sp., *Quercus* sp., *Trachycarpus* sp., are not associated with apple orchards and showed that bees went for other food sources out of the orchards once the blossom was over. In two locations (1 and 7), the *orange* pollen was dominant, this likely due to still a large amount of dandelion in the apple orchards at the time of collection. According to scientific literature in melissopalinology (Persano and Oddo Ricciardelli D’Albore, 1989) the Asteraceae T form includes echinolophate pollen grains belonging to *Taraxacum* and other genera from the Asteraceae family, which cannot be distinguished easily by light microscopy. However, from our field observations we deduced that the Asteraceae T form corresponded mainly to the genus *Taraxacum* as it was in full flowering in immediate vicinity of the bee hives during the sampling period. Furthermore, *Taraxacum* is considered a typical element of the undergrowth of apple orchards.

We detected a total of 36 substances, a lower amount than found in other studies (i.e., Mullin et al., 2010; Böhme et al., 2018). This is probably due to the lower agricultural diversity of our experimental area, where only apple trees are cultivated. Yet, we found a number of molecules higher than Stoner and Eitzer (2013) (18 molecules), Chauzat et al. (2006) (19 molecules) and close to Colwell et al. (2017) (39 molecules), whom collected pollen from more complex agricultural landscapes. The pollen from apple monoculture reached a range of pesticides comparable to that of a study comprising three different crops (Colwell et al., 2017). In our study, we did not find any pollen sample free of pesticide. As the apiaries were inside apple orchards, the contamination was expected. This finding agrees with the study of Böhme et al. (2018), which found residues in all the pollen samples from the “fruit” area (30% surface occupied by permanent vine, pome, stone, and soft fruits).

The number of fungicide residues per sample did not vary between the collections, as these substances were allowed in apple orchards also during flowering. Because of the use of most insecticides was forbidden during apple blossom, the higher number and concentration of insecticide residues found in the second collection resulted likely from the treatments applied after the end of the apple blossom. During the F collection, the *green* pollen showed a number of residues very close to that of *total* pollen, while the *orange* had a lower occurrence of both fungicides and insecticides. Nevertheless, residual concentrations at the F collection were lower than the AF collection. It is likely that these amounts were remains from the pre-floral treatments and degraded from their initial amount. In a study on residues on pollen loads (Škerl et al., 2009) reported that residues of the insecticide Diazinon reduced from 1.98 ppm 1 day after application to 0.03 ppm 10 days after (application of 15 L/ha of “Oleodiazinon”). In addition, Thiacloprid shrank from 0.09 ppm to undetectable in 6 days after treatment (application of 0.2 L/ha of “Calypso SC480”). Imidacloprid half-life was 8.2 days after foliar application (Juraske et al., 2009), and 32 days according to Vogeler et al. (1992) (but not allowed before apple blossom). Moreover, the pesticide molecules might have broken in their metabolites during the process of degradation, and then being undetected by the analyses.

The presence of pesticides also varied between pollen groups. Some of the molecules found in pollen loads from the first collection were not allowed to be sprayed during apple blossom (Imidacloprid, Chlorpyrifos-methyl, Phosmet, Etofenprox, and Flonicamid), as stated by the local guidelines (AGRIOS, 2017). The guidelines provide rules for the allowed chemicals, the number of use and the prohibition periods. However, the farmers are free to choose the treatments within this range. It was not possible then, to know exactly what pesticides were applied in the surroundings of the study sites.

Chlorpyrifos-methyl and Phosmet were found only in few locations, Etofenprox was detected at the same concentrations in two locations both in *green* and *total* pollen but Flonicamid resulted in seven locations both in *green* and *total* samples and four times in *orange* samples. We detected Imidacloprid in a *total* pollen sample during flowering, but not in the *green* pollen from the same location. As Imidacloprid was allowed only after apple blossom, this residue might come from other sources than apple flowers.

Whether these residuals belonged to treatments prior to the prohibition period, we cannot know. On the other hand, we detected only few samples with bee-friendly insecticides (Pirimicarb, Tau-fluvalinate, and Thiacloprid), allowed by the guidelines. Surprisingly, we also found residues of Chlorpyrifos-ethyl in one location during apple blossom and in two locations at the AF collection, even if the use of this pesticide was forbidden starting from spring 2017 (AGRIOS, 2017).

Application of PHQs allowed to summarize the detailed information provided by the frequency and the concentration of the residues detected in the view of honeybee toxicity.

Hazard Quotient (HQ) is a concept adopted by EU guidelines (Campbell et al., 2000) for the evaluation of side effects of chemicals on honeybees and used for the estimation of risk by pesticide exposure to honeybees (Rortais et al., 2005; Halm et al., 2006), then applied on pollen by Stoner and Eitzer (2013). The sum of the contribution of each residue provides the total PHQ. We found differences in tPHQ values between the two collection period. The pollen collected after the apple blossom had a much higher toxicological risk to honeybees, and this was indeed expected as the main treatments against aphids occurred on those weeks. However, due to the variability of the data, it was not possible to state any effect of the pollen color group on the tPHQ. While the values varied among color group in the same location, no common trends arose. Although not linked with pollen groups, this study presents, however, particularly high tPHQ values, with the majority of samples that easily exceed 40,000 after the apple blossom. Compared to previous studies, only Stoner and Eitzer (2013) had some values higher than 40,000, while in McArt et al. (2017) only Indoxacarb and Thiamethoxam had mean PHQ above 4,000. A maximum PHQ value exceeding 500 was reported only four times in Böhme et al. (2018).

Even ignoring more complex mechanisms, such as pesticide synergisms and antagonisms, the current PHQs might be of a great risk to honeybee colonies. A PHQ of 40,000 means, indeed, an assumption of 40% the LD50 in a day. At the end of this study, however, we have not been reported of any colony loss by the beekeepers. One possible answer to this survival is the social behavior of the honeybee, which, acting as a superorganism, allows them a resilience attitude to tolerate environmental stressors (Straub et al., 2015). Moreover, though the tPHQ concept comes in handy to risk estimation, it can be strongly affected by minimal variations of very toxic residuals. For instance, imidacloprid oral LD50 is 0,0039 μg/bee (US EPA ECOTOX, 2018). The highest residue we found was 180 ppb, which lead to a PHQ of 46153, 99.3% of the tPHQ of that pollen sample. Few molecules (chlorpyrifos-ethyl, chlorpyrifos-methyl, imidacloprid) influence most of the value, and minimal variation drastically change the output.

Dandelion pollen remained abundant during apple blossom, partially reducing after it but still representing a major fraction of the collection. *Taraxacum* spp. bloom usually starts few weeks before apple bloom and last through the summer (Cockfield et al., 2007). It reaches the peak bloom right before the apple full blossom, becoming an important competitor for pollinators (Free, 1968) thus decreasing but maintaining a moderate flowering until autumn. In the year of the experiment, a master thesis work (Ungerer, 2017) reported dandelion to bloom until the end of May in three locations of this study region. Dandelion is closely associated with apple orchards and very abundant on beneath (Olsen et al., 1979; Cockfield and Beers, 2008), also in the experimental locations (Ungerer, 2017, authors observation). In the experimental locations, where the absence of other crops than apple enclosed the most of the dandelion in apple orchards, this species was a reliable proxy of the pesticide treatments in orchards after the end of apple bloom. We cannot exclude a dandelion contribution from spots outside apple plantations (road edges, garden patches or ditch sides), but the overwhelming orchards surface was likely to contribute the most. It should be noticed that the local guidelines suggested mowing the orchard floor if flowers were present at the time of pesticide treatment. Regulation compliance may explain the very low amount of *orange* pollen found in few locations during the AF collection (0.5, 1.16, and 3.7%), but Ungerer (2017) also reported an average orchard floor height of 28.8 cm during the same days of the AF collection, and dandelion flowers in all the three sample sites.

While ground floor mowing may prevent bees from direct contamination during spraying, soil and leaves can absorb systemic insecticides and translocate them to nectar and pollen (Blacquiere et al., 2012) of the ground cover plants. As a result, plant with a constant flowering such as dandelion may continuously bring contaminated flowers throughout the season. The consistent amount of insecticide residues detected on the AF *orange* samples might be explained either by the lack of mowing before treatments, or the contamination of dandelion plants after flower cut, and the subsequent appearance of new flowers with pesticide residues. The latest hypothesis is supported by the high tPHQ of the *orange* portions, in spite of their low abundance in the collection. High residuals of imidacloprid were found in *orange* samples, strongly affecting their tPHQ.

After the removal of the *green* and *orange* pollen, the *leftover* represented mostly plants not associated with the apple orchard. The study of Ungerer (2017) also reported the herbaceous plants occurring in the orchard floors during the season. Combining these data with the palynological analysis of the collected pollen loads allowed understanding how much they contribute to the pollen loads. During the apple blossom, dandelion was a major fraction of the honeybee-collected pollen loads, but all the other species contribute for only 0.41%. These species (*Aegopodium podagraria* L., *Cardamine hirsuta* L., *Lamium album* L., *Ranunculus* spp., *Stellaria media* L., *Veronica persica* Poir.) are indeed reported as poorly or not attractive to honeybees (Contessi, 2016). After the apple blossom, their contribution remained very low (1.1%), dandelion was still an important portion, while most of the pollen came from ornamental or wild trees in the surrounding urban and forest area. The number of residues and the tPHQ of the *leftover* pollen groups did not differ from the *green* and the *orange* pollen groups, suggesting a pesticide contamination of the surrounding environment as high as in the apple orchards. The reasons beyond these findings may lay on pollen trans-contamination or pesticide drift. The first implies a theoretical transfer of residues between pollen loads while stored before color separation, leading to an averaging of the residues concentrations. This mechanism seemed however unlikely, as the pollen was deep-frozen after the collection and separated while still cold, remaining at room temperature only in the pollen trap drawer during the day of collection. Moreover, pollen groups from the same locations displayed consistent differences in concentrations and tPHQ values. Pesticides can reach the area outside crops by drift during and after spraying and by volatilization from soil and plant surface (Himel et al., 1990), generating pesticide-enriched rainfalls (Jensen et al., 2007), and atmospheric dust deposition (Wheatley, 1973). Spray drift is a mechanism of pesticide droplets moving through air or water (Felsot et al., 2010), and a constant concern of pesticide use (Damalas, 2015). The droplets spread according to droplet size and weather conditions, and the drift can reach a long distance when adjuvants reduce evaporation of small droplets (Bretthauer, 2011). As a result, airborne pesticide residues transport over distances of several miles may be responsible for adverse effects on non-target species (Plimmer, 1990), and the residues we found on plant pollen outside the apple orchards confirm it. Whether we do not know the relative distance of these plants from the orchards, some of them are strictly forest species (*Fraxinus ornus* L., *Quercus* spp., *Pinus* spp., *Robinia pseudoacacia* L.), most likely occurring on the fringes of the side valley woods. Their contribution to the pollen composition exceed 45% in some of the locations (2, 5, and 6) during the second collection and the tPHQ of their pollen had values equal to the *orange* or *total* ones, suggesting those areas being rich in pesticide residues as much as apple orchards.

At the best of our knowledge, it was not possible to state if the pollen from any color group posed a higher risk to the honeybees than another, nor we found any group less toxic. Each location had its own intrinsic differences between pollen groups, and the pollen collected after the flowering, when treatments were allowed again, was richer in residues and potentially more harmful than the pollen collected during the flowering. However, the variations between pollen groups in the same location clearly showed that it is actually possible to recognize the residue contamination by different pollen sources. This study also provides evidences of pesticide contamination in the surrounding environment, urban or forest, as high as in agricultural fields. Future studies that consider this approach should increase the number of locations. Moreover, the method itself can be improved, enhancing the specificity of pollen groups.

## Data Availability

The raw data supporting the conclusions of this manuscript will be made available by the authors, without undue reservation, to any qualified researcher.

## Author Contributions

SA and RF conceived and designed the study. LB managed the sample collections and part of the palynology analyses. EB and MR performed the palynology analyses on the total pollen samples. LD performed the chemical multi-residual analyses. RF performed the data collection and statistical analyses, and prepared the draft of the manuscript. SA approved the final version of the manuscript.

## Conflict of Interest Statement

The authors declare that the research was conducted in the absence of any commercial or financial relationships that could be construed as a potential conflict of interest.
